# Effectiveness Without Efficacy: Cautionary Tale from a Landmark Breast Cancer Randomized Controlled Trial

**DOI:** 10.7150/jca.79797

**Published:** 2023-01-01

**Authors:** Yu Shen, Jing Ning, Heather Y Lin, Simona F. Shaitelman, Henry M Kuerer, Isabelle Bedrosian

**Affiliations:** 1 Department of Biostatistics, The University of Texas, MD Anderson Cancer Center, Houston TX, USA.; 2 Department of Radiation Oncology, The University of Texas, MD Anderson Cancer Center, Houston TX, USA.; 3 Department of Breast Surgical Oncology, The University of Texas, MD Anderson Cancer Center, Houston TX, USA.

**Keywords:** Breast conserving therapy, Competing risks analysis, Randomized clinical trial, Total mastectomy

## Abstract

**Background:** “Old” randomized controlled trials established breast conserving therapy (BCT) and total mastectomy (TM) equivalence for treating early breast cancer, whereas recent literature report improved survival with BCT. To reconcile this, we performed a simulation study and re-analyzed B-06 trial data.

**Methods:** We estimated the distributions for overall survival (OS), cumulative incidence functions for breast-cancer-specific death (BCSD) and other causes-specific death (OCSD) by BCT and TM. The restricted mean survival time (RMST) difference and hazard ratio between the two arms were estimated. Given the estimated distributions, we simulated cause-specific death times from each arm, evaluating the power to test treatment difference in OS, BCSD, and OCSD with different sample sizes, follow-up times, and a modified setting by simulating BCT-arm OCSD times from the distribution of patients not receiving radiation.

**Results:** With 200 months follow-up, the average BCT-over-TM gain measured by RMST was 3.7 months for OS and 4.5 months for BCSD. Increasing the trial size to 5,000 per arm, there is a 79.2% chance to detect the OS benefit with RMST and 92.4% for BCSD. A nonproportional increase of OCSD in BCT compared to TM was observed after 144 months, and particularly after 200 months post treatments. When OCSD times of BCT were simulated using patients not receiving radiation, the estimated OS gain increased to 4.4 months, and the power increased to 92.2%.

**Conclusions:** The late excess other-cause-death, likely due to radiation, in the BCT arm and sample size constraints limited the power to report BCT superiority. Given radiation delivered in the era of B-06 trial, BCT and TM remain largely equivalent.

## Introduction

In the 1980s and 1990s, results from multiple randomized clinical trials changed breast cancer practice by reporting the equivalence of breast conserving therapy (BCT) and mastectomy for treatment of early stage breast cancer 1-10. The National Surgical Adjuvant Breast and Bowel Project (NSABP) B-06 trial, the largest of these randomized trials, compared lumpectomy with (or without) breast irradiation to total mastectomy (TM) for patients with tumors 4 cm or less in size. B-06 investigators have reported up to 20 years of follow-up in a series of manuscripts 1,2; no statistically significant differences were detected for the primary endpoints of overall survival (OS) and breast-cancer-specific death (BCSD) among the three treatment arms.

Recently, a growing body of literature based on cancer registry data, with cohorts in the tens of thousands, has consistently reported that early stage breast cancer patients who received BCT had better OS than those who underwent TM. Among them, Van Maaren et al. 11 showed improved OS for BCT in 37,207 patients using the Netherlands Cancer Registry of patients diagnosed between 2000 to 2004. Agarwal et al used the Surveillance, Epidemiology, and End Results database on 132,149 early stage breast cancer patients diagnosed between 1998 and 2008 and found a survival benefit favoring BCT 12. Data from the California Cancer registry of 112,154 patients diagnosed in 1990 to 2004 and the Norway Cancer Registry of 13,015 patients diagnosed in 1998 to 2008 showed an OS benefit favoring BCT 13,14. Most recently, a Swedish cohort study of 48, 986 women with T1-2N0-2 breast cancer diagnosed between 2008 to 2017 again showed better OS with BCT over TM 15.

These conflicting conclusions raise two critical questions. First, do BCT and TM provide equivalent OS when treating early stage breast cancer in the current era? Second, are the results obtained from trials in the 1970 to 1980s applicable to early stage breast cancer patients today? Multiple factors, including more personalized treatments and improvements in the delivery of radiation that reduce secondary cardiovascular toxicities, open the possibility that such differences in practice could account for the discrepancy between older trial data and more contemporary observational data. Using original data from the B-06 trial together with a simulation of the B-06 trial, we sought to provide insight into these considerations.

## Materials and methods

### Study Design, Setting, and Participants

The B-06 trial enrolled 2163 women with early stage invasive breast tumors between 1976 and 1984. Among eligible patients, 1851 accepted the assigned randomization to lumpectomy alone, TM, or BCT and had complete follow-up information up to when the trial was officially closed in 2007. The primary objective of the B-06 trial was to determine the efficacy of BCT in terms of OS and BCSD compared with TM and with lumpectomy alone 1. Details on the design of the trial have been described by the NSABP study team. In this post-hoc re-analysis, we included all 1851 eligible patients, but the efficacy analyses focused on patients randomized to the BCT (n = 628) and TM (n = 589) arms. This study was reviewed and approved by the NSABP Operations Center and the Institutional Review Board of The University of Texas MD Anderson Cancer Center.

### Statistical Analysis

OS, BCSD, and OCSD were all measured from the date of surgery. The two competing causes of death were recorded as death with evidence of breast cancer (BCSD) or death without evidence of cancer (OCSD). With a maximum of 30 years of follow-up, the Kaplan-Meier survival curves crossed around year 17 (~200 months) post-surgery (Suppl Fig 1), and the proportional hazards assumption was violated (p-value = 0.024) 16. We performed the primary analyses by truncating the follow-up data at 200 months before the two Kaplan-Meier curves started crossover; the proportional hazard assumption was not rejected (p-value=0.39). Hazard ratios estimated from the univariate Cox regression model were used to assess the difference between the two arms. To better describe the treatment effect without assuming the proportional hazards and to assess the effect over various follow-up time periods if needed, we also used the difference in restricted mean survival time (RMST) 17, which measures the area between two survival curves up to a specific follow-up time point. The RMST difference has an advantage in interpreting the treatment effect between two arms as the average OS time gain (or loss) in a specified time period.

For competing risks analyses, we estimated the cumulative incidence curve (CIC) for BCSD or OCSD and the cause-specific hazard functions by treatment group. The nonparametric cause-specific hazard function was estimated using the B-splines from the generalized linear mixed model 18. We compared the difference between CICs of two treatment arms with an HR using Gray's test 19,20, as well as the alternative to quantify the difference in BCSD or OCSD between two arms via RMST, which is the area between the CICs for each cause of death 17.

To enhance understanding of how the B-06 trial design impacts statistical power for detecting a treatment effect on the cause-specific or OS outcomes, we used a simulation framework to resample cause-specific death times based on estimators from the original B-06 data under various settings. We first estimated the nonparametric OS distributions, and CIC functions for BCSD and OCSD by accounting for competing risks and stratified by BCT and TM. We then used the aforementioned estimated distributions to simulate 5000 hypothetical trials with the two arms under several scenarios, as described next.

To investigate how follow-up duration and trial-level sample size affected the statistical power to detect OS and cause-specific survival differences, we simulated 5000 randomized trials (1:1) of BCT versus TM with a follow-up of 144 or 200 months using four different sample sizes, the actual sample size of B-06 trial, increased the sample size to 2000, 5000, or 10,000 in each arm. We defined them as **Scenario 1**. We generated death times and censoring times from the Kaplan-Meier estimator of the OS distribution 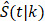 and censoring distribution 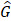
(t|k) estimated from the original B-06 trial data (*k = 1* for BCT, *k = 0* for TM). The cause-specific indicator at each death time was generated from the BC-specific incidence function, 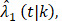
and other cause incidence function, 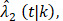
by treatment arm. We modified a simulation algorithm by Beyersmann et al 21 to generate competing risks data using the cause-specific incidence functions. Given each unique death time *Y = t*, a time-dependent binomial distribution was run to decide the cause of death. The probability of BCSD (defined as 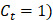 is:



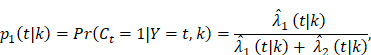



and of OCSD at *t* is, 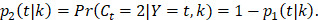 Within each treatment arm, we independently simulated the *i*-th patient's death time *Y_i_*, censoring time *U_i._* If *Y_i_ > U_i_*, the patient's death time was censored at *U_i_*; otherwise, the patient's death time *Y_i_* was recorded. The cause of death at *Y_i_* was generated from the binomial distribution with a probability of BCSD 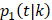, and OCSD with 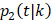.

**Figure 1:**
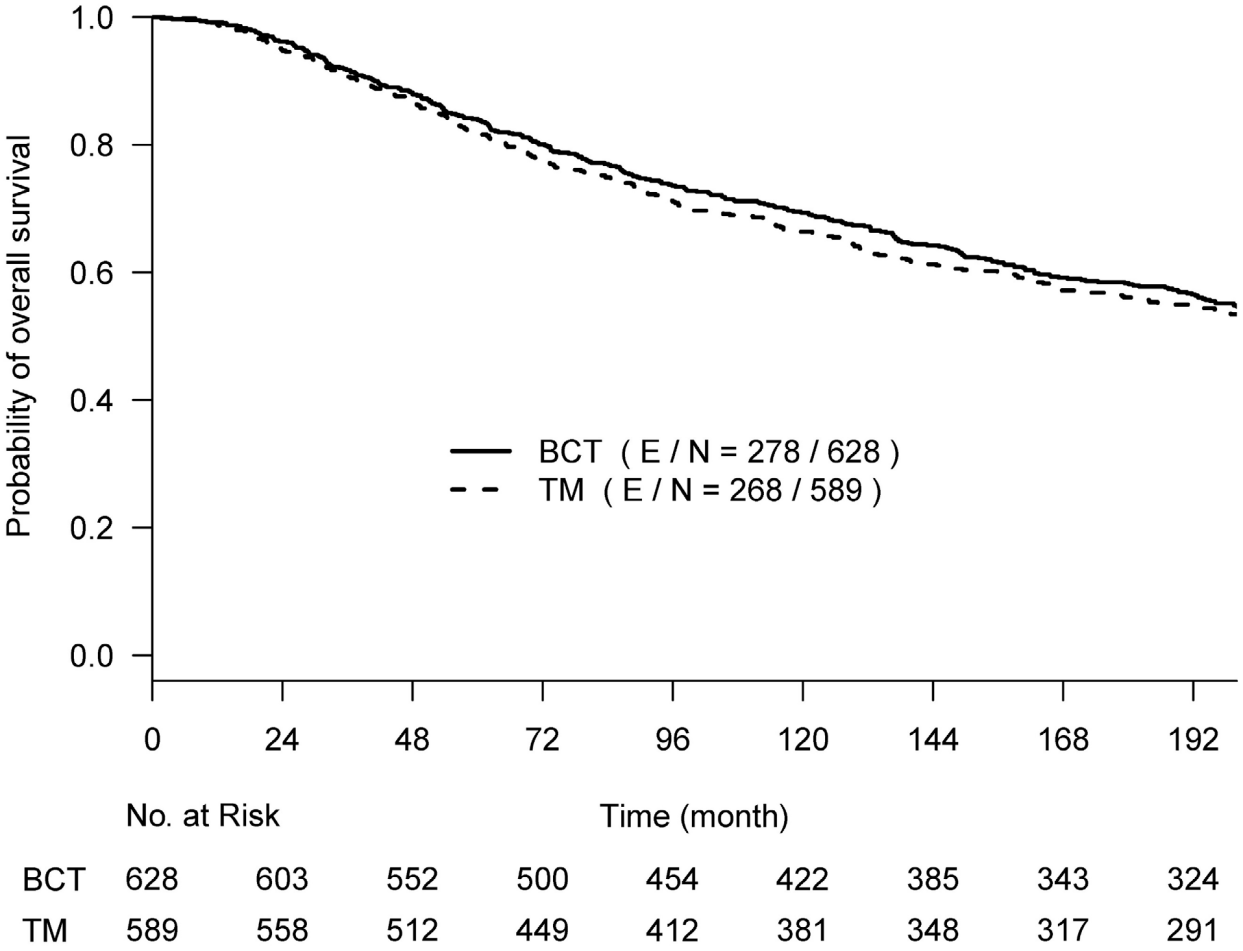
Kaplan-Meier curves of overall survival with the follow-up time truncated at 17 years post-surgery among 628 women treated with lumpectomy plus irradiation (BCT, solid line) and 589 women treated with total mastectomy (TM, dashed line).

**Figure 2:**
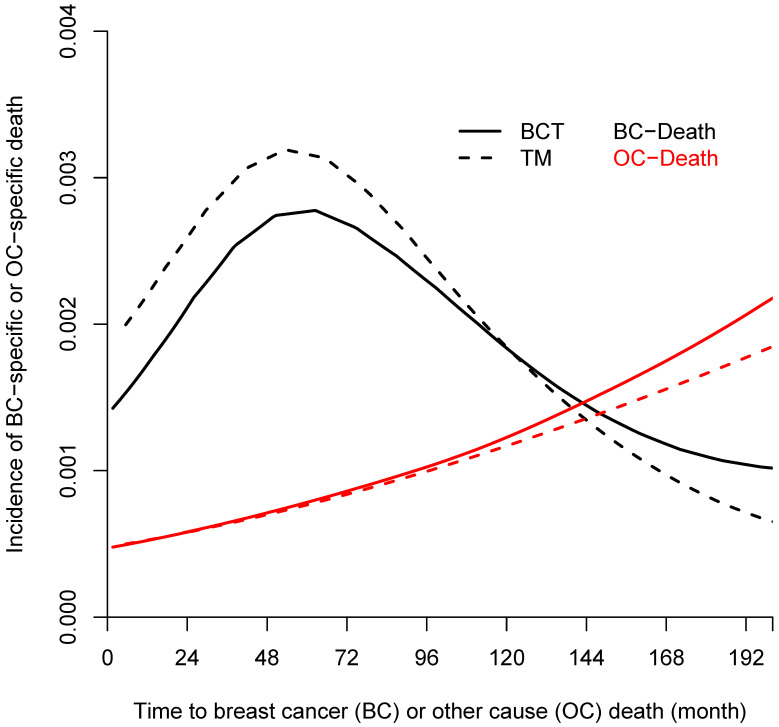
Incidence of breast-cancer-specific death (BCSD, black line) and other causes-specific death (OCSD, red line) among 628 women treated with lumpectomy plus irradiation (BCT, solid line) and 589 women treated with total mastectomy (TM, dashed line).

From the Kaplan-Meier estimators with 200 months of follow-up (Fig 1), we observed a non-proportional drop of OS probability, which corresponded to a sharp increase in the OCSD in the BCT arm beyond 144 months of follow-up (Fig 2). The observation of higher rates of OCSD later in the follow up period in the BCT arm relative to the TM arm is consistent with late toxicities of historic radiation therapy. To remove the extra, later onset OCSD from radiation in the BCT arm, we revisited this case by simulating the OCSD times for the BCT arm using the distribution from the arms in the B-06 trial that did not receive radiation. This is referred as **Scenario 2**. We simulated death time *Y_i_* in the BCT arm with the estimated all-cause hazards, using 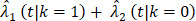, in which 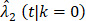 is the incidence function OCSD of the TM arm. In the BCT arm, the probability of OCSD at *t* is adjusted as:



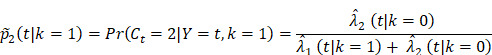



and the probability of BCSD is 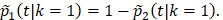
The cause-specific survival times for the TM arm were simulated the same as in **Scenario 1**. For the simulated data, we performed all the comparisons on OS, BCSD, and OCSD between BCT and TM.

Given a sharp increase in OCSD in the BCT arm beyond 12 years (144 months) of follow-up, we also performed all the comparisons by truncating the maximum follow-up time to 144 months in the both scenarios between BCT and TM, thus mitigating the effect of secondary radiation-induced toxicity on measured outcomes.

In each setting, we compared the OS between the BCT and TM arms using HR and RMST. We also performed competing risk analyses using HR and RMST to quantify treatment effects on BCSD and OCSD. For 5000 simulated trials per scenario, we summarized the percent of times to reject the null hypothesis that OS is the same, BCSD is the same, or OCSD is the same between the BCT and TM arms, respectively. We calculated the average of the estimated OS difference in RMST and HR and the power to detect the differences (p-value < 0.05) from 5000 repeated trials. The RStudio (1.3.1093) software was used for all analyses and simulations. All p-values were based on two-sided tests, and p-values < 0.05 were considered statistically significant.

## Results

### B-06 Data Re-Analysis: Comparison using hazard ratios

The primary re-analyses used the maximum follow-up data of 200 months. The estimated HR of OS between BCT and TM was 0.94 (95% CI: 0.80, 1.12), BCSD of 0.95 (95% CI: 0.86,1.05), and OCSD of 1.04 (95% CI: 0.90,1.2). The estimated smoothed cause-specific incidence functions (Fig 2) showed a higher incidence of BCSD early on for TM than BCT (within 144 months) but a decreased incidence of BCSD after 144 months for both treatment arms, corresponding to the estimated CICs (Supp Fig 2). Risk of OCSD increased over time in both arms, but for the BCT arm this increased at a higher rate than the TM arm starting from 144 months. Using data based on the follow-up of 144 months, HR of OS between BCT and TM decreased to 0.90 (0.75, 1.09). None of the comparisons were statistically significant. These data suggest that additional late effects on OCSD in the BCT arm could obscure potential OS benefit of BCT over TM.

### B-06 Data Re-Analysis: Comparison using RMST

With a follow-up time of 200 months, we obtained an RMST estimate of 150.6 (95% CI: 145.5,155.7) months in the BCT arm and 146.9 (95% CI: 141.4,152.3) months in the TM arm, which showed an average 3.7 (95% CI: -3.8,11.2; p=0.33) months OS difference for BCT vs TM. When truncated at 144 months of follow up, the estimated OS difference in RMST was 2.6 (95% CI: -2.2, 7.4; p=0.29) months for BCT vs TM. The difference of cause-specific outcome through 200 months between BCT and TM was 4.5 (95% CI: -2.7,11.8; p=0.22) months for BCSD, and -0.52 (95% CI: -5.4, 4.4; p=0.83) months for OCSD. With a follow-up of 144 months, the estimated RMST difference between the two arms was 3.1 (95% CI: -1.5, 7.7; p=0.19) months for BCSD and -0.30 (95% CI: -3.1, 2.5; p=0.83) month for OCSD.

### Simulation Scenario 1: Data simulated from original estimators of the B-06 trial

The percent of tests rejecting the null hypothesis is summarized in Table 1 by BCSD or OCSD between the two arms for different follow-up times and trial sample sizes. For Scenario 1, we simulated cause-specific death times using the estimated distributions obtained in the B-06 trial. With the B-06 trial sample size, the probability of detecting a statistically significant difference by RMST between BCT and TM was 19.5% for OS, 24.1% for BCSD, and 4.8% for OCSD; the power to detect the difference by HR between the two arms had a similar pattern as by RMST when the proportional hazards assumption was satisfied through a 144-month follow-up. It is interesting to note that the statistical power to detect OS or BCSD differences with a 200-month follow-up was much lower than that with 144-month follow-up given the same sample size. For example, the power to detect an OS difference by HR was 88.4% with a follow-up of 144 months compared to the power of 43.1% with a follow-up of 200 months given the same trial size of 5000 per arm. With 200-month follow-up, the statistical power to detect OS or BCSD differences is much higher using RMST than using HR, especially when the sample size was increased: e.g., power increased from 16.1% with the B-06 trial size to 79.2% for OS measured by RMST with a trial size of 5000, while power increased from 9.5% to 43.1% for OS measured by HR. For OCSD, the test based on RMST robustly remained between 5-8% likelihood to claim an OCSD difference regardless of trial sizes. Given the same sample size, the power to detect difference in BCSD was consistently higher than that to detect OS, which indicates that the higher OCSD for BCT offset the OS benefit of BCT. In addition, the trend of non-proportional hazards of OS between the two arms started after 144 months follow-up, and due to late excess OCSD in BCT.

### Simulation Scenario 2: Data simulated from modified estimators of the B-06 trial

The incidence trend of OCSD for BCT and TM was similar during the first decade, but the increased risk for BCT was seen after 144 months post surgery (Fig 2). To explore the long-term effect of radiation on OS, we simulated the OCSD times of the patients not receiving radiation in the B-06 trial, hypothesizing that the late effects of radiation could lead to a higher OCSD rate, thus impacting OS in the BCT arm. As shown in Table 1, the estimated OS gain measured by the RMST increased from 3.7 (in Scenario 1) to 4.4 months (in Scenario 2) during 200 months of follow-up, and with a trial sample size of 5000, the power to detect the RMST difference in OS was 79.2% (Scenario 1), however this increased to 92% for Scenario 2. The probabilities to detect OS difference either by HR or RMST were much higher than those of Scenario 1 given the same sample size, but to a lesser degree for 144 months of follow-up. For BCSD outcome, an average of 4.8 months gain in BCT over TM arm was estimated through 200 months. The power to detect the difference of HR and RMST in OCSD between the two arms remained under 9.2% for all sample sizes, which is in contrast to those of Scenario 1 with 200 months of follow-up. As shown in Fig 2, the BCSD rate of the BCT arm was lower than that of the TM arm during the first decade, and the OCSD rate of BCT arm started increasing after 144 months. Therefore, with follow-up terminated at 144 months, the probabilities to detect OS, BCSD, and OCSD under Scenario 2 were similar to those under Scenario 1.

## Discussion

Randomized clinical trials in the 1970s and 1980s played a critical role in establishing the equivalence of BCT and mastectomy in treating early stage breast cancer patients, though more contemporary Cancer Registry data consistently report a survival benefit favoring BCT. Our re-analysis of the B-06 data provides clues for this discrepancy: an unanticipated non-proportional increase due to excess other causes death after the first decade in the BCT arm, coupled by the lack of statistical power with the B-06 trial size, may have contributed to the failure to find a small long term OS benefit for BCT over TM.

Our re-analysis under a resampling and simulation framework 22 demonstrated late effects on OCSD in the BCT arm, likely due to radiation, and that controlling for these late negative effects increased improvement in long term survival in favor of BCT. However, our findings also demonstrated that the probability of detecting this small to moderate benefit in survival was low with the B-06 trial sample size, requiring a sample size of 5000 to achieve a power of 80% or above, a number far in excess of any of the randomized trials that compared BCT to TM. Later onset OCSD not only offset the OS benefit of BCT, but also led to a nonproportional hazard between the two arms. In such a setting, the conventional test using hazard ratio had lower power, while differences measured by RMST were more robust and sensitive. Our re-analysis detected a small difference in OS between BCT and TM with increased trial sizes, however this difference was far smaller than those reported in more recent observational cohorts.

Our findings also underscore that all RCTs comparing BCT and TM outcomes were not originally designed to test equivalence or non-inferiority between BCT and TM. It is not ideal to use such RCT findings to establish noninferiority 23,24. Logistically, not being able to reject the hypothesis that BCT is the same as TM in OS (as designed in a superiority trial) does not provide the desired evidence to establish equivalence of the two arms with pre-defined type I and type II error rates. Such a trial could be severely under powered to test equivalence or non-inferiority in OS or cumulative incidence of BCSD between the BCT and TM groups.

Our observation that OCSD was higher after a decade in BCT patients suggests late effects related to radiation. This is in line with multiple reports noting long-term cardiac mortality from radiation, especially for treatment delivered in the era of the B-06 trial 25. The Early Breast Cancer Trialists Group meta-analysis of 40 RCTs with a combined sample size of nearly 20,000 early breast cancer patients (all treated before 1990) suggested that the moderate improvement in BCSD was counterbalanced by increased mortality from other causes 26. This meta-analysis observed the excess OCSD rate mainly attributed from vascular deaths, perhaps due to inadvertent irradiation of the coronary, carotid, or other major arteries. The introduction of CT in radiotherapy in the early 1990s enabled robust calculations of radiation dose exposure of the cardiac structures. This, together with the development of innovative techniques such as prone positioning and deep inspiration breath hold 27,28, and discrete CT-derived metrics driving modern breast radiotherapy treatment planning 29-31 have resulted in concerted efforts to minimize lung and heart radiation doses. The improvement of radiotherapy after the 1990s could contribute to the improved OS benefit observed from more contemporary observational data.

The landmark NSABP B-06 trial was performed before standardized pathologic margin assessment, routine screening mammography and breast imaging to select patients for lumpectomy, and prior to the use of modern improved conformal radiotherapy and systemic therapies. Taken together with data from other randomized trials and subsequent single center/multicenter studies over the past 50 years this trial was pivotal in establishing breast conserving therapy as a potential preferred and safe management approach for breast cancer in the past and in the present clinical environment. It should be noted that this trial was the forefather that laid the foundation for the potential of eliminating all breast cancer surgery for low-risk invasive breast cancer which is currently being studied and has demonstrated very early promising results 32.

Revisiting the B-06 trial within the context of current understanding of trial design and treatment effects provides clinicians with reassurance that BCT and mastectomy remain fairly similar oncologic outcome. The decision to undergo breast conserving therapy versus mastectomy with or without reconstruction is a very personal decision for patients with breast cancer. There are some patients where mastectomy will be an optimal choice based on clinical criteria and patient choice. Notwithstanding, among patients who meet clinical criteria for breast conserving therapy or mastectomy with or without reconstruction the ideal treatment approach needs to weigh patient choice and open multidisciplinary discussion.

## Limitations

Our findings do not consider the possibility that the outcomes of BCT and mastectomy patients may be different if considered within the context of subtype of breast cancer.

## Figures and Tables

**Table 1:** Power to detect difference in overall survival, breast cancer-specific death and other causes-specific death between breast conserving therapy and total mastectomy arms under various simulation scenarios with 5000 repeats

			Truncate follow-up at 144 months				Truncate follow-up at 200 months					
	OS^a^		BCSD^b^		OCSD^c^		OS		BCSD		OCSD	
	HR^*^	RMST^†^	HR	RMST	HR	RMST	HR	RMST	HR	RMST	HR	RMST
***Scenario 1***												
**Estimated Difference**	0.90	2.62	0.94	3.1	.99	-0.3	0.94	3.7	0.95	4.5	1.04	-0.52
Actual size of B-06	19.6	19.5	24.2	24.1	4.8	4.8	9.5	16.1	15.8	21.4	7.0	5.0
N1=N2=2000	51.4	50.5	62.0	61.1	5.0	5.0	20.4	41.9	40.4	56.4	14.5	5.8
N1=N2=5000	88.4	86.2	95.0	94.5	6.4	5.4	43.1	79.2	76.4	92.4	28.3	6.6
N1=N2=10000	99.5	99.2	99.9	99.9	8.3	5.9	71.4	97.4	96.7	99.7	49.4	8.2
***Scenario 2***												
**Estimated Difference**	0.88	2.86	0.87	3.1	1.05	-.03	0.92	4.4	0.89	4.8	0.98	0.15
Actual size of B-06	26.1	22.1	29.4	26.3	4.7	5.4	15.8	21.4	17.9	25.7	5.5	5.0
N1=N2=2000	65.9	55.6	70.9	67.3	5.0	5.2	38.5	55.7	46.3	65.9	5.7	5.6
N1=N2=5000	96.2	91.2	97.7	96.4	5.9	5.5	76.7	92.0	84.3	95.7	6.7	5.9
N1=N2=10000	100	99.7	99.9	100	6.8	4.9	96.8	99.7	99.1	100	9.2	6.2

^a^ Overall survival; ^b^ Breast cancer-specific death; ^c^ Other causes-specific death; * Hazard ratio; ^†^ restricted mean survival time.
